# 

**Published:** 1886-02

**Authors:** 


WHOLE NUMBER, CCLXXX. FEBRUARY, 1886. NUMBER 7. VOLUME XXVI. 25

### THE BUFFALO Medical and Surgical Journal

ESTABLISHED IN YEAR 1844.

LIBRARY INDEXED S.G.O.

INDEX MEDICUS

**EDITORS:**

**THOS. LOTHROP, M. D.** **A. R. DAVIDSON, M. D.**

ASSOCIATE EDITORS:

FLOYD S. CREGO, M. D. CARLTON C. FREDERICK, M. D.

FRED. R. CAMPBELL, M. D. HERBERT MICKLE, M. D.

EDWARD B. ANGELL, M. D., Rochester, N. Y.

Entered at the Post-Office at Buffalo, N. Y., as Second-class Mail Matter.

